# Plasma Metabolic and Lipidomic Fingerprinting of Individuals with Increased Intestinal Permeability

**DOI:** 10.3390/metabo12040302

**Published:** 2022-03-29

**Authors:** Rohan M. Shah, Snehal R. Jadhav, Laura Phan, Kelton Tremellen, Cuong D. Tran, David J. Beale

**Affiliations:** 1 Land and Water, Commonwealth Scientific and Industrial Research Organization, Ecosciences Precinct, Dutton Park, QLD 4102, Australia; rshah@swin.edu.au; 2 Department of Chemistry and Biotechnology, School of Science, Swinburne University of Technology, Melbourne, VIC 3122, Australia; 3 Consumer-Analytical-Safety-Sensory (CASS) Food Research Centre, Deakin University, Melbourne, VIC 3125, Australia; snehal.jadhav@deakin.edu.au; 4 Clinical & Health Sciences, Faculty of Health Sciences, The University of Adelaide, Adelaide, SA 5000, Australia; laura.phan@unisa.edu.au; 5 Department of Obstetrics Gynaecology and Reproductive Medicine, Flinders University, Bedford Park, Adelaide, SA 5001, Australia; kelton.tremellen@flinders.edu.au; 6 Health and Biosecurity, Commonwealth Scientific and Industrial Research Organization, Gate 13 Kintore Ave, Adelaide, SA 5000, Australia; cuong.tran@csiro.au; 7 School of Medical Sciences, Faculty of Health Sciences, The University of Adelaide, Adelaide, SA 5000, Australia

**Keywords:** intestinal permeability, lactulose-to-rhamnose ratio, metabolomics, lipidomics

## Abstract

The dual-sugar intestinal permeability test is a commonly used test to assess changes in gut barrier function. However, it does not identify functional changes and the exact mechanism of damage caused by the increased intestinal permeability. This study aims to explore the application of untargeted metabolomics and lipidomics to identify markers of increased intestinal permeability. Fifty fasting male participants (18–50 years) attended a single visit to conduct the following procedures: assessment of anthropometric measures, assessment of gastrointestinal symptoms, intestinal permeability test, and assessment of blood samples 90 min post-administration of the intestinal permeability test. Rhamnose and lactulose were analysed using gas chromatography-mass spectrometry (GC-MS). Untargeted polar metabolites and lipidomics were assessed by liquid chromatography quadrupole time-of-flight mass spectrometry (LC-QToF MS). There was an elevated lactulose/rhamnose ratio in 27 subjects, indicating increased permeability compared to the remaining 23 control subjects. There were no significant differences between groups in characteristics such as age, body mass index (BMI), weight, height, and waist conference. Fourteen metabolites from the targeted metabolomics data were identified as statistically significant in the plasma samples from intestinal permeability subjects. The untargeted metabolomics and lipidomics analyses yielded fifteen and fifty-one statistically significant features, respectively. Individuals with slightly elevated intestinal permeability had altered energy, nucleotide, and amino acid metabolism, in addition to increased glutamine levels. Whether these biomarkers may be used to predict the early onset of leaky gut warrants further investigation.

## 1. Introduction

The gut functions as an important barrier between the environment and the host. The intestine is the physical barrier separating the gut lumen from the inner host [1,2]. The intestinal barrier constitution is quite complex and consists of a mucus layer, gut microbes, antimicrobial peptides, secretory IgA, vascular cells, cytokines, immune cells, and a layer of epithelial cells linked by tight junction proteins [3,4,5].

The intestinal barrier selectively facilitates the intake of nutrients and water, while preventing the entry of foreign antigens or harmful substances into the body [1,6]. In the case of dysfunctional intestinal permeability, commonly known as leaky gut (LG) whereby the barrier loses its ability to control the influx of substances from the gut into the body; this can result in microbes, viruses, and other toxic metabolites gaining easy access from the gut lumen to the blood and lymphatic system and other organs in the body, leading to local and systemic inflammation [2,3,4,5,7].

LG has been associated with the healthy ageing process, and in the case of infections from pathogenic bacteria, cardiovascular diseases (CVD), coeliac disease, inflammatory bowel disease (IBD), non-alcoholic liver disease, colon cancer, obesity, Parkinson’s disease, type 2 diabetes, neuroimmune disorders, and allergies [2,3,7,8]. Western-type diets rich in fats and refined sugars can alter the intestinal microbiome, in turn causing LG. Adverse reactions to foods, chronic alcohol use, patients using nonsteroidal anti-inflammatory drugs (NSAIDs), systemic inflammation, psychological stress, and other lifestyle factors can also result in increased intestinal permeability [7].

One of the conventional methods to detect dysfunctional intestinal permeability (IP) of the small intestine is by using the dual-sugar assay, consisting of the oral ingestion of non-digestible sugars lactulose (L, molecular weight = 342 Da) and rhamnose (R, molecular weight = 164), or mannitol (M, molecular weight = 182 Da). The former is a relatively large molecule, and can only be absorbed via the paracellular pathway of translocation when the intestinal barrier is compromised. In contrast, rhamnose/mannitol are smaller molecules that can easily diffuse through the barrier via the transcellular pathway and are affected by gastric dilation, motility, the presence of bacteria, and renal function [1,3,7,9]. When the barrier is severely damaged, the permeability of rhamnose/mannitol is decreased. Hence, determining the L/R or L/M ratio serves as an indicator of IP [1]. Traditionally, this assay has been performed by analyzing urine excretions over a 6-h period from the time of ingestion [3]. However, impaired renal function can alter the serum levels of L/R and a recent improvement in this assay has been to directly measure L/R levels in the blood, invalidating the need for adjustment according to renal function [10]. Furthermore, this direct serum assessment of L/R levels speeds up the patient monitoring time from 6 h down to only 90 min, improving patient acceptance.

Although the dual-sugar test is used popularly, it has some limitations. The presence of baseline mannitol/rhamnose (pre-ingestion of sugar samples) in some individuals, most probably due to food contaminants or from the use of medication, has been found to affect final L/R or L/M ratios [3]. The test has a lower sensitivity, is time-consuming, and can lead to false negatives [7,11,12]. Lastly, individual differences in physiology, metabolism, and diet have been found to alter the test results [3]. More importantly, it does not let us identify functional changes and the exact mechanism of damage caused by the increased permeability [6].

Alteration of the intestinal microbiome (otherwise known as ‘gut dysbiosis’) plays an important role in the manifestation of increased intestinal permeability (IP). It is hypothesized that gut dysbiosis can impact body glucose and lipid metabolism in the host, possibly through alterations in insulin sensitivity [13]. Thus, a study of the metabolome (small metabolites ≤ 1.5 kDa) and the lipidome (lipid profile) can offer valuable insights in understanding increased IP and the molecular pathways that impact the microbiome-host interactions during dysbiosis [14]. Such approaches can also aid in the identification of biomarkers for the early onset of IP.

The current study is a proof-of-concept investigation to explore the application of untargeted metabolomics and lipidomics to identify markers of increased IP. A follow-up study would be to confirm these findings in participants with confirmed leaky gut. Post-validation of these markers may assist in the development of rapid diagnostic tests for detecting the early onset of IP.

## 2. Results and Discussion

### 2.1. Clinical Characteristics

**Figure 1:**
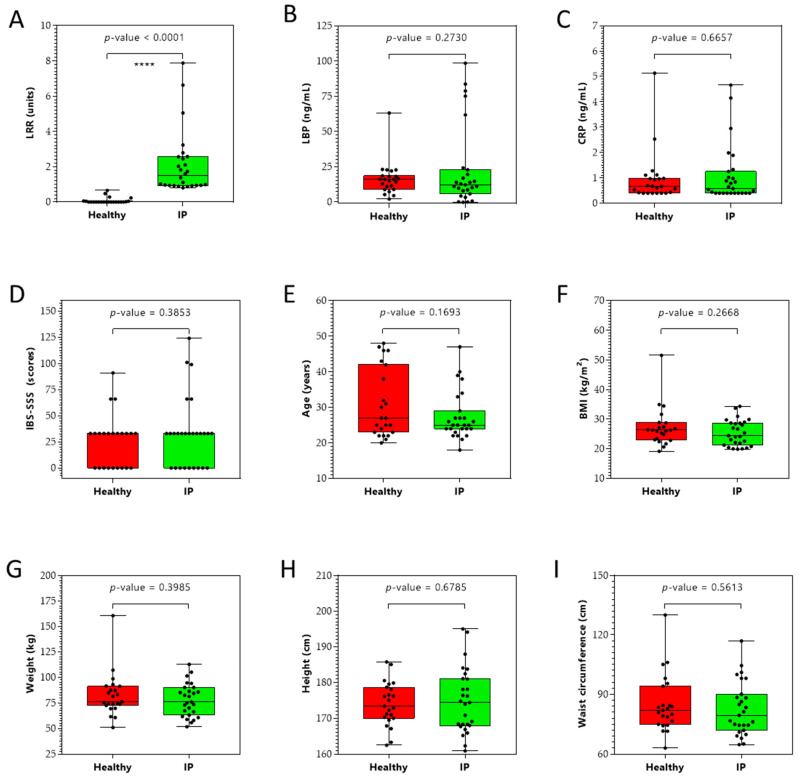
Clinical (panels **A**–**D**), demographic (panel **E**), and anthropometric (panels **F**–**I**) characteristics of study participants (*n* = 50). (**A**) Lactulose/rhamnose ratio (LRR), (**B**) lipopolysaccharide binding protein (LBP) levels, (**C**) C-reactive protein (CRP) levels, (**D**) irritable bowel syndrome severity scoring system (IBS-SSS), (**E**) age, (**F**) body mass index (BMI), (**G**) weight, (**H**) height, and (**I**) waist circumference of healthy and increased intestinal permeability (IP) study participants. **** *p* < 0.0001.

The IP test was performed at baseline and 90 min post-administration of sugar drink on 50 subjects to measure the lactulose/rhamnose ratio (LRR). This test is a sugar absorption test with two orally administered sugars, lactulose and rhamnose, which are unmetabolizable. These sugars are passively absorbed water-soluble compounds that measure the intactness of the tight junctions. Lactulose (a disaccharide) is absorbed in very small amounts through the paracellular pathway via tight junctions, and rhamnose (a monosaccharide) is easily transported via the transcellular pathway. In the case of increased IP (or relative leakiness), the tight junctions allow more lactulose to pass the intestinal barrier and enter blood circulation before excretion via urine. The results (Figure 1A) showed an elevated LRR in 27 subjects, indicating increased IP; these subjects were assigned as increased IP subjects. The remaining 23 subjects were assigned as healthy (control) subjects. No correlation was observed between the LRR ratio and leaky gut inflammatory markers lipopolysaccharide binding protein (LBP) and C-reactive protein (CRP). In addition, no correlation was observed between the irritable bowel syndrome severity scoring system (IBS-SSS) scores and the LRR ratio. These findings suggest that IP subjects did not have LG but were most likely exhibiting an early onset of LG [15,16]. The baseline clinical characteristics of the study sample are shown in Figure 1. A total of 50 male adults participated in the study: 27 IP subjects and 23 healthy subjects. There were no significant (*p*-value > 0.05) differences between groups in the clinical characteristics (Figure 1B–D).

### 2.2. Targeted Metabolomics

**Figure 2:**
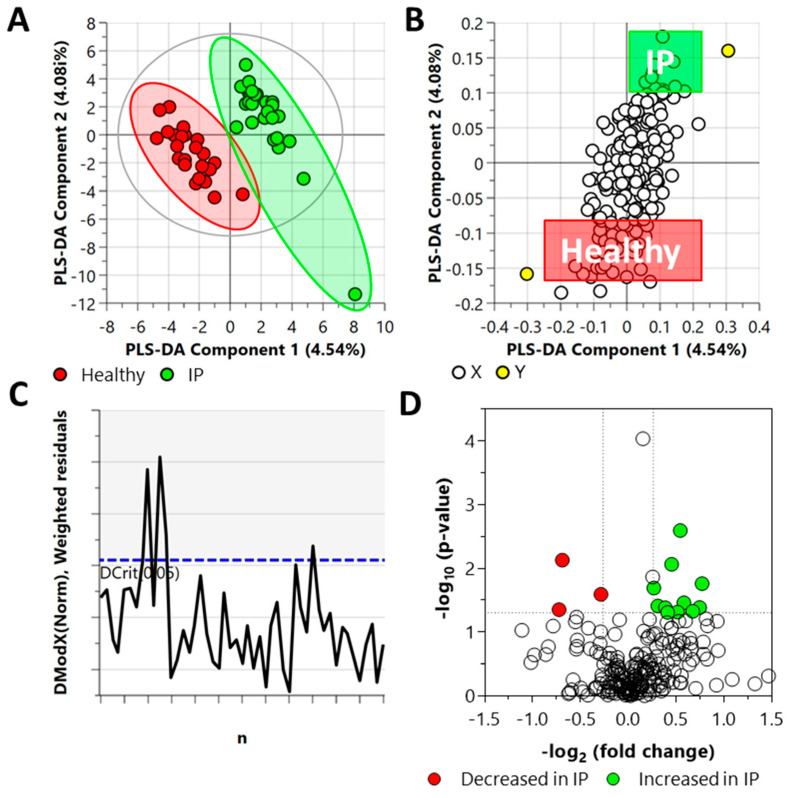
Distinct metabolic profiles of plasma samples in increased IP subjects compared with the healthy subjects, as revealed by the Partial Least Square Discriminant Analysis (PLS-DA) (panels **A**–**C**) and fold change (FC) analysis (panel **D**). (**A**) PLS-DA scatter plot, (**B**) PLS-DA loading plot, (**C**) distance of observation plot, and (**D**) volcano plot of plasma samples collected from study participants. R^2^X (cum) = 0.0862, R^2^Y (cum) = 0.947, Q^2^ = 0.372, DCrit (blue line in panel **C**) = 1.222. *Note: The ellipse presented in panel A represents Hotelling’s T2 confidence limit (95%). The colored circles in panel A represent each analyzed sample, while the yellow-colored circles in panel B indicate the average group position for each sample cluster, with the white circles representing the distribution of metabolite features between these groups. The colored circles in panel D represent significant metabolites in IP subjects compared with the healthy subjects*.

A targeted metabolomics approach to analyze 219 central carbon metabolism intermediates in plasma of increased IP and healthy subjects was performed. Figure 2 illustrates the global view of increased IP metabolic characteristics. The partial least squares discriminant analysis (PLS-DA) analysis of the targeted acquired data (Figure 2, Q^2^ = 37.2%) resulted in better model separation compared to the principal component analysis (PCA) model created from the plasma samples (Supplementary Figure S1, Q^2^ = 0.56%).

The DModX plot indicated that three samples exceeded the Dcrit threshold for rejecting a sample. The threshold for a moderate outlier is considered when the sample DmodX value is twice the Dcrit at *p*-value = 0.05, which, in this instance, was 2.444 (Dcrit = 1.222). This ultimately resulted in no data points being removed from the analysis. Cross-validation of this model resulted in a *p*-value of 0.0073 (Supplementary Figure S1).

Fold change (FC) analysis of the targeted acquired data identified 14 metabolites as statistically significant (FC ≥ 1.2 or FC ≤ 0.83, and *p*-value ≤ 0.05); eleven of them increased and three decreased in the plasma samples from IP subjects (Figure 2) as compared to the healthy subjects. The elevated metabolites included L-asparagine, DL-glyceraldehyde 3-phosphate, N-carbamyl-L-glutamic acid, L-glutamine, 5-deoxy-5-(methylthio)adenosine, homocitrate, adenosine 3–5-cyclic monophosphate, D-ribose 5-phosphate, inosine 5-monophosphate, uridine 5-diphosphate, and S-5-adenosyl-L-homocysteine. The metabolites that decreased are malonic acid, glyceric acid, and alpha-D-mannose 1-phosphate.

### 2.3. Untargeted Metabolomics and Lipidomics

**Figure 3:**
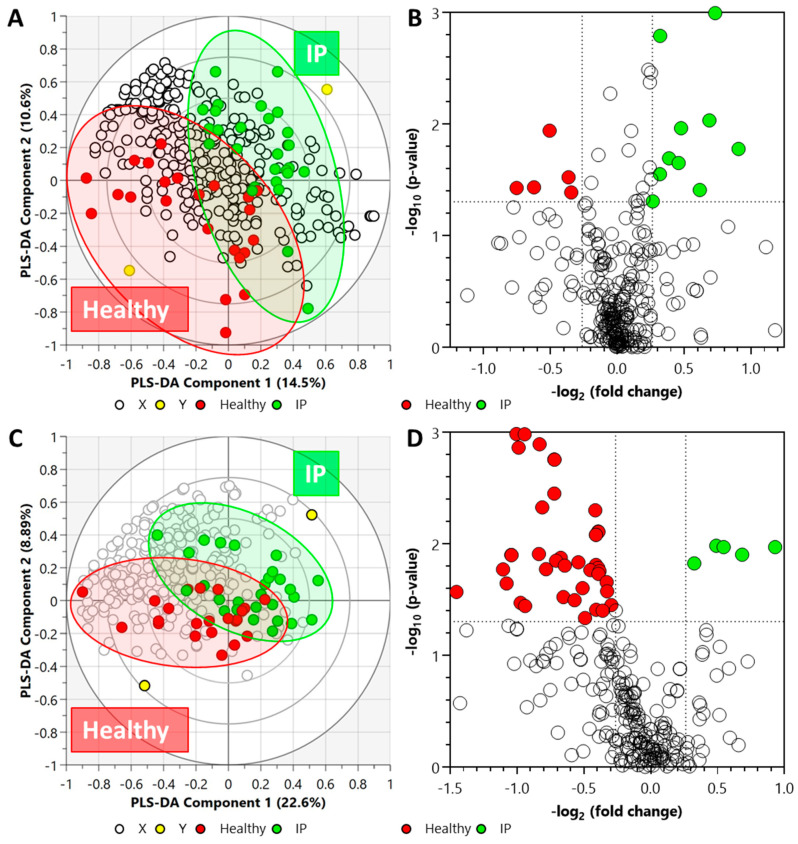
Biplot overview (panels **A**,**C**) of LC-QToF MS acquired data set and associated global view (panels **B**,**D**) of the significant compounds. (Panels **A**,**B**) metabolite data set from plasma samples (R^2^X = 0.358, R^2^Y = 0.934, Q^2^ = 0.355), and (panels **C**,**D**) lipid data set from plasma samples (R^2^X = 0.572, R^2^Y = 0.833, and Q^2^ = 0.0746). *Note: In panels (**A**,**C**), the ellipses on the biplots represent the 100%, 75%, and 50% correlation coefficient for measured metabolites. The colored circles represent each analyzed sample, while the yellow-colored circles indicate the averaged group position for each sample cluster, and the white circles represent the distribution of LC-QToF MS detected metabolites between increased intestinal permeability (IP) and healthy groups. The colored circles in panel (**D**) represent significant metabolites in IP subjects compared with the healthy subjects*.

In the LC-QToF MS metabolomics data set, after annotation, 296 metabolites were detected based on tandem MS (MS/MS) spectra, of which 150 were identified in the plasma samples. The PLS-DA analysis of the acquired metabolite data from the plasma samples is illustrated in Figure 3A.

Cross-validation of this model resulted in a *p*-value of 0.0096 (Supplementary Figure S2). Fifteen metabolites were detected as statistically significant in the plasma samples from IP subjects; ten (of which three were identified) were higher, and five (of which four were identified) were lower (Figure 3B) as compared to the healthy subjects. The list of significant metabolites and associated FC values and *p*-values are illustrated in Supplementary Table S1.

In the LC-QToF MS lipidomics data set, after annotation, 316 lipids were identified in the plasma samples based on MS/MS spectra. After annotation, 316 lipids were identified in the plasma samples. Figure 3C illustrates the PLS-DA analysis of the acquired lipid data from the plasma samples. The plasma lipidomics PLS-DA data set was found to be statistically non-significant (*p*-value = 0.9093) (Supplementary Figure S2). In the plasma samples, a total of 51 lipids were identified to be statistically significant; forty-six decreased and five increased in the elevated IP subjects. Whilst acylcarnitine and ether-linked phosphatidylethanolamines elevated, lipids from other classes, including ceramide non-hydroxy fatty acid-sphingosines, lysophosphatidylcholines, phosphatidylcholines, phosphatidylethanolamines, and triacylglycerols, declined in increased IP subjects (Figure 3D). The list of significant lipids and associated FC values and *p*-values are illustrated in Supplementary Table S2.

### 2.4. Chemical and Pathway Enrichment Analysis

We performed a chemical similarity enrichment analysis using ChemRICH [17] on the central carbon metabolism data set acquired from the plasma samples. This provided chemical class-based information of significantly altered metabolites in each sample type analyzed. ChemRICH identifies highly impacted compound classes through the generation of metabolite clusters based on chemical similarity and ontologies that are not defined by organism-specific metabolic pathways, which can be inherently flawed [17]. ChemRICH analysis does not rely upon background databases for statistical calculations. The most impacted compound clusters (*p* ≤ 0.1) are summarized in Supplementary Table S3. These included basic amino acids, sugar acids, adenosine, and tricarboxylic acids. Several metabolites from the FC analysis were classified within these chemical clusters.

**Figure 4:**
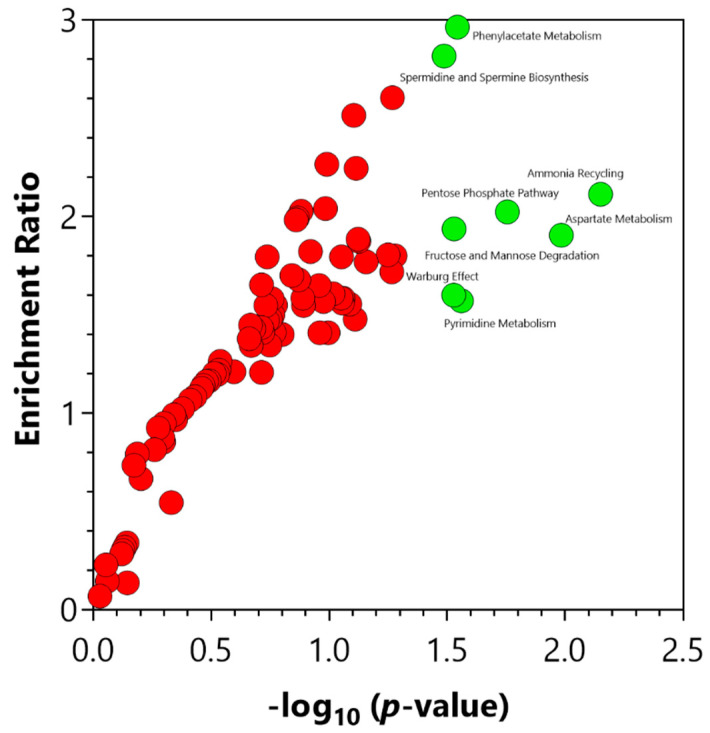
Significant pathways identified via the enrichment analysis of the central carbon metabolism intermediates using MetaboAnalyst 5.0 (Enrichment Analysis Toolbox, Quebec, Canada). Pathways annotated with red circles were enriched but found not to be significant. Pathways annotated with green circles were significantly (*p*-value ≤ 0.05) enriched. The significantly enriched pathways are illustrated in Supplementary Table S4.

The enrichment analysis of the central carbon metabolism intermediates in the plasma samples indicated a significant (*p*-value ≤ 0.05) change in several metabolic pathways (Figure 4) in the increased IP subjects. These pathways included ammonia recycling, aspartate metabolism, pentose phosphate pathway, pyrimidine metabolism, phenylacetate metabolism, fructose and mannose degradation, Warburg effect, and spermidine and spermine biosynthesis (Supplementary Table S4).

### 2.5. Biomarker Analysis

**Figure 5:**
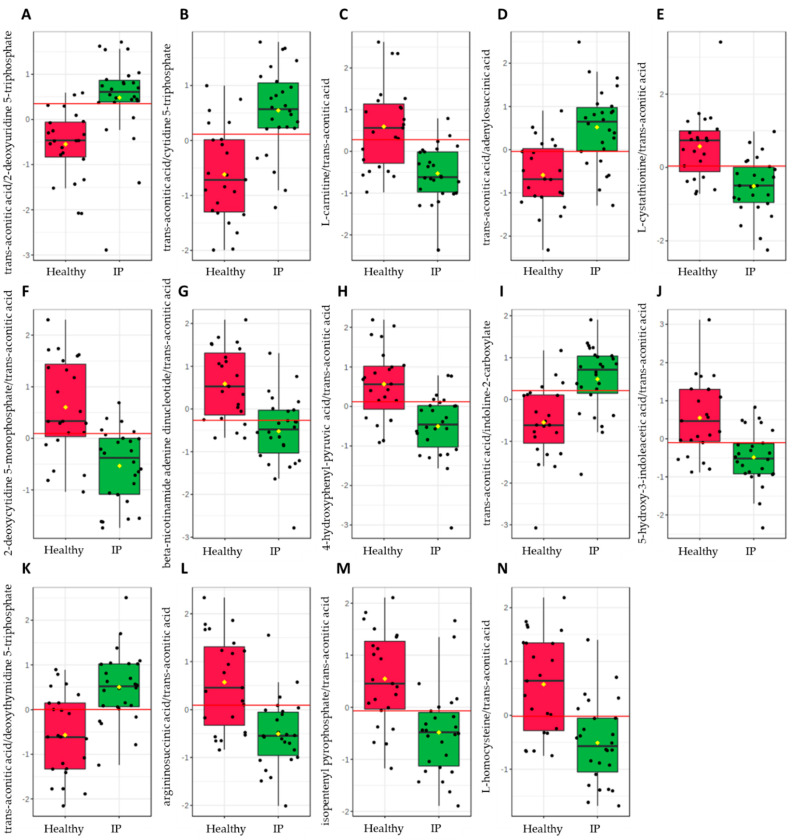
Significant metabolite ratios (*p*-value ≤ 0.05) with a good diagnostic value (AUC ≥ 0.8) in increased intestinal permeability (IP) subjects from the plasma samples identified via the biomarker analysis of the central carbon metabolism intermediates using MetaboAnalyst 5.0 (Biomarker Analysis Toolbox, Quebec, Canada). (**A**) trans-aconitic acid/2-deoxyuridine 5-triphosphate, (**B**) trans-aconitic acid/cytidine 5-triphosphate, (**C**) L-carnitine/trans-aconitic acid, (**D**) trans-aconitic acid/adenylosuccinic acid, (**E**) L-cystathionine/trans-aconitic acid, (**F**) 2-deoxycytidine 5-monophosphate/trans-aconitic acid, (**G**) beta-nicotinamide adenine dinucleotide/trans-aconitic acid, (**H**) 4-hydroxyphenyl-pyruvic acid/trans-aconitic acid, (**I**) trans-aconitic acid/indoline-2-carboxylate, (**J**) 5-hydroxy-3-indoleacetic acid/trans-aconitic acid, (**K**) trans-aconitic acid/deoxythymidine 5-triphosphate, (**L**) argininosuccinic acid/trans-aconitic acid, (**M**) isopentenyl pyrophosphate/trans-aconitic acid, and (**N**) L-homocysteine/trans-aconitic acid.

To evaluate the diagnostic accuracy of these differential metabolites in plasma for increased IP subjects, a predictive model for subject classification was constructed using central carbon metabolism intermediates. Overall, 24 metabolites or metabolite ratios were statistically significant (*p*-value ≤ 0.05) and had a potentially useful diagnostic value with the area under the curve (AUC) above 0.7 in plasma samples (Supplementary Table S5). Of these, 14 metabolite ratios had a good diagnostic value with the AUC above 0.8 (Figure 5), and included trans-aconitic acid/2-deoxyuridine 5-triphosphate (AUC = 0.8679), trans-aconitic acid/cytidine 5-triphosphate (AUC = 0.8374), L-carnitine/trans-aconitic acid (AUC = 0.8311), trans-aconitic acid/adenylosuccinic acid (AUC = 0.8261), L-cystathionine/trans-aconitic acid (AUC = 0.8244), 2-deoxycytidine 5-monophosphate/trans-aconitic acid (AUC = 0.8211), beta-nicotinamide adenine dinucleotide/trans-aconitic acid (AUC = 0.8194), 4-hydroxyphenyl-pyruvic acid/trans-aconitic acid (AUC = 0.8161), trans-aconitic acid/indoline-2-carboxylate (AUC = 0.8161), 5-hydroxy-3-indoleacetic acid/trans-aconitic acid (AUC = 0.8060), trans-aconitic acid/deoxythymidine 5-triphosphate (AUC = 0.8060), argininosuccinic acid/trans-aconitic acid (AUC = 0.8027), isopentenyl pyrophosphate/trans-aconitic acid (AUC = 0.8027), and L-homocysteine/trans-aconitic acid (AUC = 0.8010).

A similar predictive model for subject classification was constructed using the LC-QToF MS acquired metabolite and lipid data sets. From the LC-QtoF MS acquired metabolite data set, 24 statistically significant (*p*-value ≤ 0.05) metabolites or metabolite ratios with a potentially useful diagnostic value (AUC > 0.7) in plasma samples (Supplementary Table S6) were obtained. Of these, 10 metabolite ratios had a good diagnostic value (AUC ≥ 0.8). These included unknown_102/2-methoxy-3-(1-methylpropyl)pyrazine (AUC = 0.8470), unknown_107/2-methoxy-3-(1-methylpropyl)pyrazine (AUC = 0.8325), unknown_13/benzylazanium (AUC = 0.8293), unknown_107/pirimicarb (AUC = 0.8213), 2,4-diaminotoluene/vanillylamine (AUC = 0.8197), unknown_40/vanillylamine (AUC = 0.8084), unknown_35/unknown_107 (AUC = 0.8035), unknown_59/unknown_107 (AUC = 0.8035), unknown_107/cinnamyl cinnamate (AUC = 0.8019), and 11-amino-undecanoic acid/vanillylamine (AUC = 0.8019).

On the other hand, the lipid data set revealed 44 statistically significant (*p*-value ≤ 0.05) lipids or lipid ratios with a potentially useful diagnostic value (AUC ≥ 0.7) in plasma samples (Supplementary Table S7). Eighteen of these had a good diagnostic value (AUC > 0.8), which included Cer_NS d18:2_24:0/TG 15:0_16:0_18:2 (AUC = 0.8451), Cer_NS d18:2_24:0/TG 15:0_16:1_18:2 (AUC = 0.8451), Cer_NS d18:1_24:1/TG 15:0_16:0_18:2 (AUC = 0.8434), ACar 12:1/SM d36:2 (AUC = 0.8384), Cer_NS d18:1_24:1/TG 15:0_16:1_18:2 (AUC = 0.8384), ACar 12:1/TG 15:0_16:1_18:2 (AUC = 0.8350), TG 15:0_16:0_18:2/TG 16:0_18:1_18:2 (AUC = 0.8300), TG 15:0_16:0_18:2/TG 52:3 (AUC = 0.8300), ACar 12:1/TG 15:0_16:0_18:2 (AUC = 0.8283), ACar 12:1/LPC 15:0/0:0 (AUC = 0.8199), ACar 10:0/TG 15:0_16:0_18:2 (AUC = 0.8182), ACar 12:1/PC 14:0_16:0 (AUC = 0.8148), TG 15:0_16:0_18:1/TG 16:0_18:1_18:2 (AUC = 0.8081), TG 15:0_16:0_18:1/TG 52:3 (AUC = 0.8081), TG 16:0_17:0_18:1/TG 16:0_18:1_18:2 (AUC = 0.8064), TG 16:0_17:0_18:1/TG 52:3 (AUC = 0.8064), ACar 12:1/TG 15:0_16:0_18:1 (AUC = 0.8047), and TG 51:3/TG 54:4 (AUC = 0.8014).

### 2.6. Pathway Mapping

**Figure 6:**
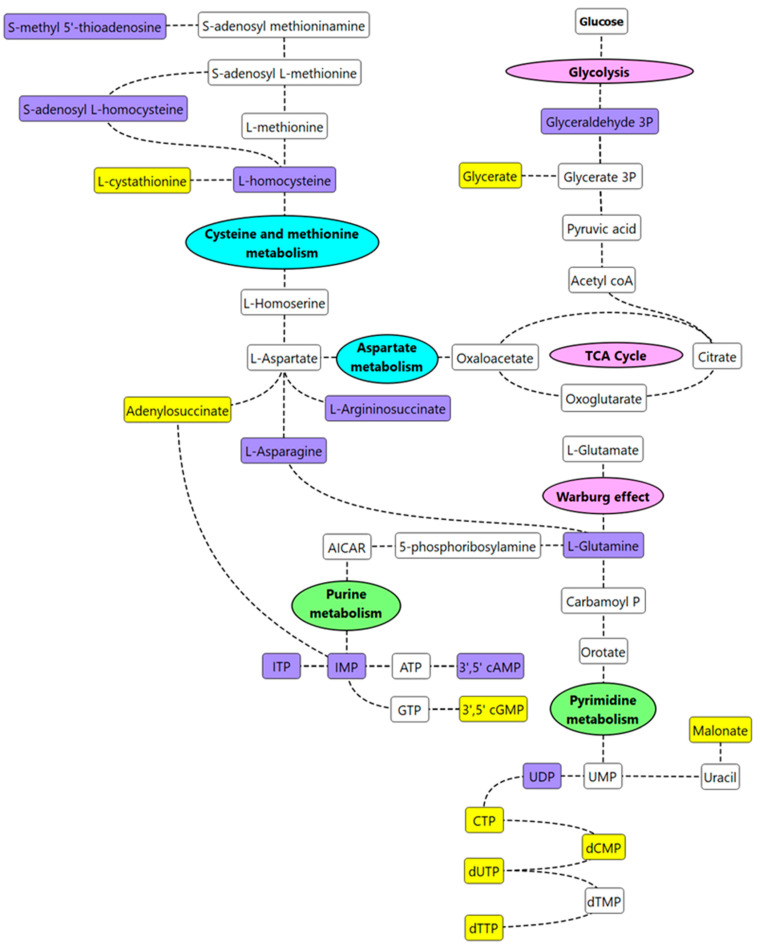
Curated metabolic pathway of key metabolism changes in IP subjects. Note, **purple-colored** metabolites are significant metabolites/putative biomarkers that are higher in IP subjects vs. healthy subjects; **yellow-colored** metabolites are significant metabolites/putative biomarkers that are lower in IP subjects vs. healthy subjects. Key metabolic pathways of interest identified from the pathway enrichment and impact analysis are annotated in **pink** (energy metabolism), **green** (nucleotide metabolism), or **blue** (amino acid metabolism).

As a means of synthesizing these findings further, a curated metabolic pathway of the significant metabolites (*p* ≤ 0.05, FC ≥ 1.2 or ≥ 0.8) and putative biomarkers were generated (Figure 6).

Increased concentrations of L-homocysteine (FC = 1.2), S-adenosylhomocysteine (FC = 1.3), and decreased concentrations of cystathionine (FC = 0.7) were observed in increased IP subjects suggesting that cysteine/methionine metabolism was most likely impacted by increased IP. Homocysteine is a metabolic intermediate formed by the demethylation of methionine. Methionine, homocysteine, and taurine are generally considered to play an important role in intestinal health [18]. High levels of homocysteine and its precursor S-adenosyl L-homocysteine have previously been reported in intestinal inflammation [19,20]. We hypothesize that homocysteine is a biomarker of increased IP and subsequent metabolic inflammation. Eventually, such metabolic inflammation and altered lipid metabolism, insulin resistance, and glycaemic control may be predisposing individuals to pathologies such as atherosclerosis and cardiovascular disease. Vitamin B supplementation is a recommended treatment for patients with hyperhomocysteinemia [21]; perhaps investigating these individuals for increased IP and if found abnormal, improving the overall gut health, may be a more preventative approach. Elevated levels of other amino acids such as L-asparagine (FC = 1.5) and L-glutamine (FC = 1.2) were also observed in increased IP subjects when compared to the healthy group. Asparagine can form aspartate via deamination reactions, and transamination of aspartate can eventually result in glutamine synthesis [22]. L-asparagine has recently been suggested to play a role in mitigating lipopolysaccharide-induced intestinal damage [22]. Glutamine, the most abundant amino acid in plasma, plays a critical role in maintaining IP; supplementation of glutamine in the diet has been observed to improve intestinal barrier function [23]. The findings from this study seem contradictory to some reports in the literature that correlate low levels of glutamine with increased IP [24,25]. We hypothesize that both aspartate and glutamate are most likely contributing to the healing of the intestinal barrier in increased IP subjects. However, this needs further investigation. In addition, nucleotide metabolism was affected in increased IP subjects with an overall decrease in metabolites linked to the pyrimidine metabolism pathway. This was in congruence with the findings of an independent study that correlated stress with an increased IP [24]. Low levels of nucleotides in the diet, in the case of mice, have been found to have an unfavourable effect on gut health; supplementation via oral administration has been associated with an overall decrease in the translocation of intraluminal microbes in serum, improving intestinal injury [26].

## 3. Materials and Methods

### 3.1. Study Design and Ethics

This was a single-arm, observational case-control study under the “Periodontitis, intestinal permeability, and testosterone (PIPT)” study approved by the University of South Australia Human Research Ethics Committee (Ethics Protocol #202371). This study aimed to analyse periodontal or gastrointestinal disease metabolic endotoxemia and altered male steroid hormone profiles.

### 3.2. Study Participants

Men between the ages of 18–50 (*n* = 50) were enrolled in this study. Participants arrived fasted to the University of South Australia Clinical Trial Facility for a single, 3-h visit.

**Inclusion Criteria**: Eligibility criteria included men aged between 18 and 50 years old with at least 24 natural teeth and who could provide informed consent.

**Exclusion Criteria**: Participants were ineligible if they were exposed to narcotic use, nicotine use, excessive alcohol consumption (>4 standard drinks/day), special diet, e.g., vegan, antibiotics within the last month, irritable bowel syndrome, autoimmune disease; medication: immunosuppressive therapy, e.g., prednisolone, antibiotics, androgen treatments, e.g., DHEA, testosterone therapy; supplements: probiotic supplements, fish oil; lifestyle: excessive exercise, smoker, excessive ROH (> 40 g/day), special diet (i.e., vegetarian/ vegan paleo, etc.); diseases: Hep B/C/HIV or chronic infective conditions, insulin-dependent diabetes, pathological hyperlipidemia (+lipid nephrosis or acute pancreatitis if accompanied by hyperlipidemia), pulmonary disease, anemia, or blood coagulation disorders.

### 3.3. Study Protocol

On arrival, an initial blood sample (20 mL, baseline) was collected from each participant by venipuncture. Participants were administered a dual-sugar solution containing 100 mL water, 5 g lactulose, and 1 g rhamnose in order to measure intestinal permeability. 1.5 h post-dual-sugar administration, another blood sample (20 mL) was obtained from each participant by venipuncture. The blood was tested for markers of increased IP. Other data including height, weight, body fat percentage, body mass index (BMI), and waist circumference were also measured.

### 3.4. Intestinal Permeability Test

The permeability of the intestine was assessed by a dual-sugar test as validated by van Wijck et al. [27,28]. Briefly, two blood samples (20 mL), before (baseline) and after administration of the water solution containing the two sugars, were collected to measure IP. Sugars (lactulose and rhamnose) were analyzed by gas chromatography with a mass spectrometry detector (described later). IP was measured by calculating the lactulose-to-rhamnose ratio (LRR) in plasma after 1.5 h.

### 3.5. Analysis of Rhamnose and Lactulose by GC-MS

An aliquot of plasma was passed through an EMR Captiva Lipid removal cartridge (96-well plate format, Agilent Technologies, Mulgrave, Victoria, Australia), as per the manufacturer’s instructions, on a Bravo Metabolomics Workbench (Agilent Technologies, Mulgrave, Victoria, Australia). The samples were then transferred to high recovery GC vials, spiked with ^13^C_2_ succinic acid (1 mg mL^−1^, Cambridge Isotope Laboratories, Tewksbury, MA, USA), and dried in a CentriVap DNA Vacuum Concentrator at 210 g and 30 °C (Labconco, Kansas City, MO, USA). The dried samples were then capped with PTFE/Silicon/PTFE sandwich septa in a magnetic crimp cap before being transferred to a GC-MS for analysis. The samples were derivatised ‘in-time’, followed by a 1-h holding time, and injection (1 µL) into an Agilent 7890B gas chromatography system with 5977B mass spectrometry detector (GC-MSD), as per the conditions previously reported [29,30]. Lactulose and rhamnose were monitored by selective ion monitoring (SIM) of two abundant TMS derivatised fragmentation ions, as determined using authentic standards. The acquired data were then processed using MassHunter Quantitative Analysis Workstation (Build 10.0.707.0, Agilent Technologies, Santa Clara, CA, USA). Rhamnose and lactulose concentrations were determined from a three-point calibration curve (50 μM, 125 μM, and 250 μM). The method detection limits for rhamnose and lactulose were 0.05 and 0.04 µM, respectively. The relative standard deviations (RSD%) of authentic rhamnose (50 µM) and lactulose (25 µM) were 0.64 and 8.52, respectively. The RSD% of the spiked internal standard ^13^C_2_ succinic acid was 6.2%.

### 3.6. Metabolite and Lipid Sample Extraction

Samples were extracted as previously published in Beale et al. [31]. Briefly, the metabolite and lipid extracts were separated via the Captiva EMR-Lipid plate (96-well, 1 mL, 40 mg, Agilent, Mulgrave, Australia). A series of blanks and mixed QC standards were prepared in the same way, without biological material; pooled biological quality control (PBQC) samples were prepared by combining 10-microlitre aliquots from each biological sample. The metabolite fraction was dried and reconstituted in 50 µL Water:MeOH (4:1, *v*/*v*). Lipids were eluted off the Captiva plate into high recovery glass vials with 500 µL of DCM:MeOH (1:2, *v*/*v*) before being dried and reconstituted in 50 µL BuOH:MeOH (1:1, *v*/*v*). Internal standard set #1 comprised 100 ppb of L-phenylalanine (1-^13^C) and L-glutamine (amide-^15^N); Internal standard set #2 comprised 200 ppb of succinic Acid (1,4-^13^C_2_), glycine (1-^13^C), L-aspartic acid (^13^C_4_), and L-valine (1-^13^C). The residual relative standard deviation (RDS%) of the internal standards ranged from 2.04 to 7.64%.

### 3.7. Central Carbon Metabolism Metabolomics (LC-QqQ-MS)

Central carbon metabolism metabolites were analyzed using an Agilent 6470 liquid chromatography triple quadrupole mass spectrometer (LC-QqQ-MS) coupled with an Agilent Infinity II ultra-high-performance liquid chromatography (UHPLC) system (Agilent Technologies, Santa Clara, CA, USA) as per Beale et al. [31], and after Gyawali et al. [32].

### 3.8. Untargeted Polar Metabolites and Non-Polar Lipids (LC–QToF-MS)

Untargeted polar metabolites and nonpolar lipids were analyzed using an Agilent 6546 liquid chromatography time-of-flight mass spectrometer (LC-QToF) with an Agilent Jet Stream source coupled to an Agilent Infinity II UHPLC system (Agilent Technologies, Santa Clara, CA, USA), as previously published [31]. Collected metabolite data were processed using MassHunter Profinder software (Version 10.0, Agilent Technologies, Santa Clara, CA, USA), normalized to IS, and putatively identified against the Agilent METLIN Metabolite PCDL (G6825-90008, Agilent Technologies, Santa Clara, CA, USA) and a curated in-house PCDL based on retention time (±0.15 min), precursor ions, MSMS spectra and a library threshold score of more than 80%. For the acquired lipid data, auto MSMS data on polled PBQC samples were obtained at collisions of 20 eV and 35 eV. The acquired MSMS lipid data were analysed using the Agilent Lipid Annotator tool (V1.0; Santa Clara, CA, USA) which assigned isometric structures based on MSMS fragmentation patterns. Annotated lipids were then curated into a PCDL, which was used to identify lipids within the remaining analyzed samples with retention time thresholds (±0.15 min), MSMS spectra, and a library threshold score of more than 80%.

### 3.9. Serum and Plasma Biomarkers

Assessment of the end-result of leaky gut, the passage of bacterial endotoxin into circulation (metabolic endotoxaemia), was performed by analysing lipopolysaccharide binding protein (LBP) levels. LBP levels were determined via sandwich ELISA (Hycult Biotech, Uden, The Netherlands) according to manufacturer’s instructions. The measurable concentration range was 4.4–50 µg/mL, with no potential cross-reactivity with other peptides, proteins, or species. Plasma LBP intra-assay precision was 2.9% at 5.7 µg/mL, 0.1% at 7.2 µg/mL, and 3.7% at 10.4 µg/mL. Inter-assay precision was 1.4% at 5.9 µg/mL, 1.6% at 7.3 µg/mL, and 9.2 at 11.0 µg/mL. Absorbance was read at 450 nm (Thermo Fisher Scientific, Waltham, MA, USA).

High sensitivity C-reactive protein (hs-CRP) levels were determined via an immunoturbidimetric assay (ITA) (Roche Diagnostics). Serum was combined with buffer solution, incubated at 37 °C, then combined with CRP antibody-coated reagent. A spectrophotometer filter set to 546 nm (Ortho Clinical Diagnostics, NJ, USA) determined turbidity, and absorbance was measured against a standard curve. The measurable range of serum CRP was 0.15–20.0 mg/mL, with respective intra-assay variations of 4.0% and 0.9% at concentrations of 3.44 mg/L and 9.14 mg/L. Inter-assay variations were 6.8% and 3.8% at concentrations of 3.06 mg/L and 1.00 mg/L, respectively.

### 3.10. Assessment of Gastrointestinal Symptoms

Gastrointestinal health status was determined by the modified Irritable Bowel Syndrome Severity Scoring System (IBS-SSS) [33].

### 3.11. Statistical Analysis and Data Integration

The metabolomics and lipidomic data were subjected to further statistical analysis using multivariate statistics. The data were first imported, matched by sample identifiers (metadata), and log-transformed in order to normalize the data using SIMCA 16.02 (MKS Data Analytics Solutions, Uméa, Sweden). Partial Least Square Discriminant Analysis (PLS-DA) was performed by finding successive orthogonal components from the cohort-specific data sets with maximum squared covariance and was subsequently used to identify the common relationships among the multiple data sets.

MetaboAnalyst 5.0 (Xia Lab, McGill University, Quebec, QC, Canada) was used for metabolic pathway analysis [34]. Metabolites and lipids with Benjamini–Hochberg adjusted p-value of ≤0.05 and fold changes (FC) of ≤0.83 (downward regulation) or ≥1.2 (upward regulation) were statistically significant. Chemical clusters based on structural similarity were created for metabolic examination using the ChemRICH analysis [17].

## 4. Conclusions

In the current study, an IP test based on an analysis of rhamnose and lactulose was conducted on 50 male participants aged 18–50 years. The increased IP subjects were differentially separated from the healthy individuals. The metabolite and lipid data sets from plasma samples of healthy and increased IP subjects were analysed by GC-MS and LC-QToF-MS. The present study demonstrated that healthy individuals with slightly elevated IP have altered energy, nucleotide, and amino acid metabolism. Considering that individuals with increased permeability were not found to exhibit clinical markers (LBP and CRP) of LG, the authors hypothesize that the cohort is most likely exhibiting markers of a predisposition to LG. More specifically, increased glutamine levels in increased IP individuals suggests that the rise in glutamine may be a positive compensatory action by the body to avoid the onset of LG. The authors hypothesize that in conditions that impair the body’s ability to generate glutamine, or from reduced dietary intake of protein (glutamine), these individuals may continue to be predisposed to LG. Whether more specific changes in energy, nucleotide and amino acid metabolism may be used to predict early onset in increased IP warrants further investigation.

## Data Availability

The data presented in this study are available on request from the corresponding author. The data are not publicly available due to intellectual property restrictions.

## Supplementary Materials

The following supporting information can be downloaded at: https://www.mdpi.com/article/10.3390/metabo12040302/s1, Figure S1: Principal component analysis (PCA) scores plot and PLS-DA cross-validation scores plot of the targeted central carbon metabolism metabolites in plasma samples; Figure S2: Cross-validation (CV) scores plot of PLS-DA model generated from untargeted metabolomics and lipidomics data; Table S1: Significant metabolites (FC ≥ 1.2 or FC ≤ 0.83 and *p* ≤ 0.05) in the plasma from the increased IP subjects using the untargeted metabolomics approach; Table S2: Significant lipids (FC ≥ 1.2 or FC ≤ 0.83 and *p* ≤ 0.05) in the plasma from the increased IP subjects using the untargeted lipidomics approach; Table S3: Identified significant metabolite clusters (*p* ≤ 0.1) in the plasma from the increased IP subjects using the central carbon metabolism metabolite data set; Table S4: Enrichment pathway analysis in the plasma from increased IP subjects using the central carbon metabolism intermediates; Table S5: Metabolites with good diagnostic value in increased IP and healthy subjects identified via biomarker analysis of plasma samples; Table S6: LC-QToF MS acquired metabolites with good diagnostic value in increased IP and healthy subjects identified via biomarker analysis of plasma samples; Table S7: LC-QToF MS acquired lipids with good diagnostic value in increased IP and healthy subjects identified via biomarker analysis of plasma samples.
