# Supplementary material for: Plasma Metabolic and Lipidomic Fingerprinting of Individuals with Increased Intestinal Permeability
Source: Metabolites. 2022 Mar 29;12(4):302. doi: 10.3390/metabo12040302 (PMC9026773; doi:10.3390/metabo12040302)
Supplement: Supplementary file 1 [file metabolites-12-00302-s001.zip › metabolites-1648852-supplementary.pdf]

metabolites

MDPI

Article

Plasma Metabolic and Lipidomic Fingerprinting of Individuals with Increased Intestinal Permeability

Rohan M. Shah [1,2], Snehal R. Jadhav [3], Laura Phan [4], Kelton Tremellen [5], Cuong D. Tran [6,7], and David J. Beale [1,*]

Supplementary Material

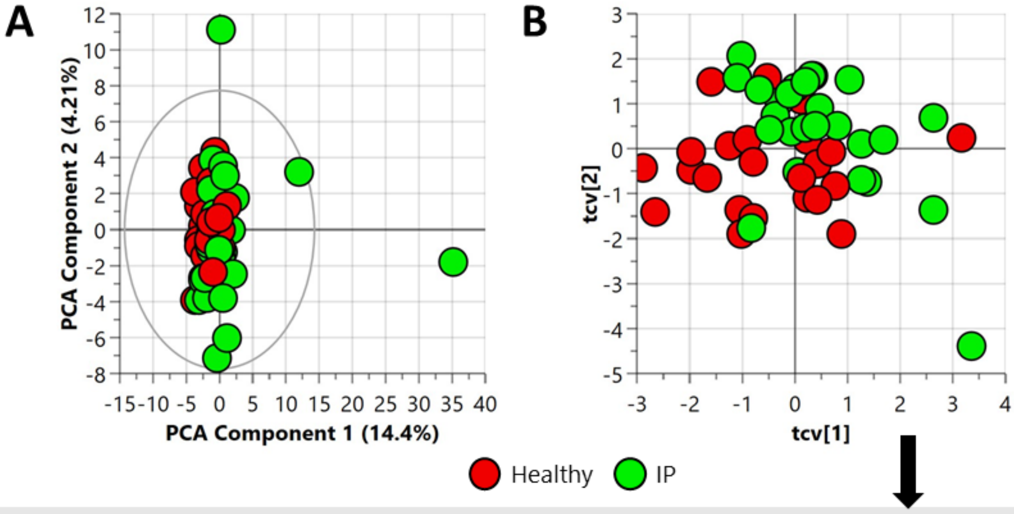

PLS-DA (Figure 1)	SS	DF	MS	F	p	SD
Total corr.	48	48	1			1
Regression	12.843	4	3.21076	4.01837	0.00729344	1.79186
Residual	35.157	44	0.799022			0.89388

Supplementary Figure S1. A. Principal component analysis (PCA) scores plot and B. PLS-DA cross-validation scores plot of the targeted central carbon metabolism metabolites in plasma samples (R^2X (cum) = 0.186, Q^2 = 0.00558). *Note: The ellipse presented in panel A and panel B represents Hotelling's T2 confidence limit (95%). The colored circles in panel A represent each analyzed sample. The table indicates cross-validation (CV)-ANOVA of the PLS-DA model.*

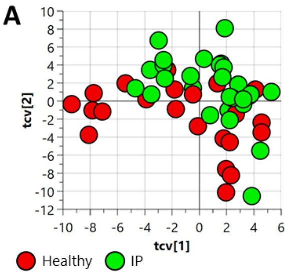

Biplot (Figure 3A)	SS	DF	MS	F	p	SD
Total corr.	49	49	1			1
Regression	18.1011	8	2.26264	3.0023	0.009573	1.50421
Residual	30.8989	41	0.753631			0.868119

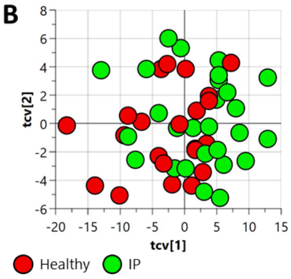

Biplot (Figure 3C)	SS	DF	MS	F	p	SD
Total corr.	48	48	1			1
Regression	1.05881	4	0.264702	0.248116	0.909272	0.514492
Residual	46.9412	44	1.06685			1.03288

Supplementary Figure S2. Cross-validation (CV) scores plot of PLS-DA model generated from untargeted metabolomics and lipidomics data. (A) Metabolite dataset from plasma samples and (B) Lipid dataset from plasma samples. *Note: The colored circles in panel A and panel B represent each analyzed sample. The tables indicate cross-validation (CV)-ANOVA of the corresponding PLS-DA models.*

Supplementary Table S1. Significant metabolites ($FC \geq 1.2$ or $FC \leq 0.83$ and $p \leq 0.05$) in the plasma from the IP subjects using the untargeted metabolomics approach.

Metabolite	FC	p-value	Upregulated or Downregulated
Unknown_107	1.66	0.0010	Up
Unknown_13	1.25	0.0016	Up
Unknown_140	1.61	0.0093	Up
Vanillylamine	1.39	0.0110	Up
Unknown_40	0.70	0.0115	Down
Unknown_8	1.88	0.0168	Up
(S)-3-Octanol glucoside	1.31	0.0204	Up
Unknown_115	1.37	0.0225	Up
Unknown_61	1.25	0.0283	Up
Pirimicarb	0.78	0.0301	Down
11-amino-undecanoic acid	0.65	0.0371	Down
Isoamyl nitrite	0.59	0.0375	Down
Unknown_11	1.53	0.0392	Up
2-Methoxy-3-(1-methylpropyl)pyrazine	0.79	0.0412	Down
Methylconiine	1.20	0.0495	Up

FC: Fold change

Supplementary Table S2. Significant lipids (FC ≥ 1.2 or FC ≤ 0.83 and p ≤ 0.05) in the plasma from the IP subjects using the untargeted lipidomics approach.

Lipid	Lipid class	FC	p-value	Upregulated or Downregulated
ACar 10:0	Acylcarnitine	1.61	0.0126	Up
ACar 10:1	Acylcarnitine	1.91	0.0108	Up
ACar 12:1	Acylcarnitine	1.41	0.0105	Up
Cer_NS d19:1/22:0	Ceramide non-hydroxy fatty acid - sphingosines	0.82	0.0357	Down
EtherPE 16:1e/22:4	Ether-linked phosphatidylethanolamines	1.26	0.0151	Up
LPC 0:0/14:0	Lysophosphatidylcholines	0.76	0.0155	Down
LPC 0:0/15:0	Lysophosphatidylcholines	0.8	0.0222	Down
LPC 14:0/0:0	Lysophosphatidylcholines	0.77	0.0187	Down
LPC 15:0/0:0	Lysophosphatidylcholines	0.76	0.0051	Down
PC 14:0/14:0	Phosphatidylcholines	0.62	0.0143	Down
PC 14:0/16:0	Phosphatidylcholines	0.69	0.0147	Down
PC 16:0/16:1	Phosphatidylcholines	0.56	0.0124	Down
PC 17:0/18:1	Phosphatidylcholines	0.64	0.0302	Down
PC 17:0/20:4	Phosphatidylcholines	0.68	0.0322	Down
PC 18:0/18:1	Phosphatidylcholines	0.77	0.0165	Down
PC 18:0/20:5	Phosphatidylcholines	0.72	0.0466	Down
PC 31:1	Phosphatidylcholines	0.53	0.0363	Down
PC 33:1	Phosphatidylcholines	0.61	0.0036	Down
PE 16:0/18:2	Phosphatidylethanolamines	0.74	0.0173	Down
PE 16:0/20:4	Phosphatidylethanolamines	0.65	0.0158	Down
PE 18:0/20:4	Phosphatidylethanolamines	1.46	0.0108	Up
PE 18:0/22:6	Phosphatidylethanolamines	0.47	0.0170	Down
TG 12:0/12:0/18:1	Triacylglycerols	0.16	0.0208	Down
TG 12:0/14:0/18:1	Triacylglycerols	0.37	0.0271	Down
TG 12:0/15:0/18:2	Triacylglycerols	0.71	0.0251	Down
TG 12:0/16:0/18:1	Triacylglycerols	0.49	0.0127	Down
TG 12:0/16:1/18:1	Triacylglycerols	0.48	0.0228	Down
TG 12:0/18:1/18:2	Triacylglycerols	0.59	0.0170	Down
TG 14:0/16:0/16:0	Triacylglycerols	0.63	0.0134	Down
TG 14:0/16:0/18:1	Triacylglycerols	0.76	0.0393	Down
TG 15:0/16:0/18:1	Triacylglycerols	0.53	0.0011	Down
TG 15:0/16:0/18:2	Triacylglycerols	0.53	0.0006	Down
TG 15:0/16:1/18:2	Triacylglycerols	0.57	0.0013	Down
TG 15:0/18:1/18:2	Triacylglycerols	0.76	0.0084	Down
TG 16:0/16:1/18:1	Triacylglycerols	0.77	0.0078	Down
TG 16:0/17:0/18:1	Triacylglycerols	0.5	0.0011	Down
TG 16:0/17:1/18:1	Triacylglycerols	0.61	0.0018	Down
TG 17:0/18:1/18:2	Triacylglycerols	0.8	0.0267	Down
TG 18:0/18:0/18:1	Triacylglycerols	0.51	0.0341	Down
TG 42:1	Triacylglycerols	0.16	0.0157	Down
TG 44:1	Triacylglycerols	0.37	0.0271	Down
TG 46:1	Triacylglycerols	0.49	0.0127	Down
TG 46:2	Triacylglycerols	0.48	0.0230	Down
TG 48:1	Triacylglycerols	0.76	0.0392	Down
TG 48:2	Triacylglycerols	0.58	0.0048	Down
TG 50:2	Triacylglycerols	0.77	0.0079	Down
TG 50:3	Triacylglycerols	0.77	0.0177	Down
TG 51:1	Triacylglycerols	0.51	0.0014	Down
TG 51:2	Triacylglycerols	0.61	0.0018	Down
TG 51:3	Triacylglycerols	0.76	0.0084	Down
TG 54:7	Triacylglycerols	0.78	0.0399	Down

FC: Fold change

Supplementary Table S3. Identified significant metabolite clusters (p ≤ 0.1) in the plasma from the IP subjects using the central carbon metabolism metabolite dataset.

Cluster name	Cluster size	p-value	FDR	Metabolites	Altered metabolites
Sugar Acids	5	0.004	0.17	2-phosphoglyceric acid<br>**Glyceric acid**<br>D-gluconic acid<br>Galactonic acid<br>Glucoheptonic acid	2
Tricarboxylic acids	5	0.023	0.50	trans-aconitic acid<br>**Homocitrate**<br>DL-isocitric acid<br>Citric acid<br>cis-aconitic acid	2
Amino acids (Basic)	3	0.045	0.65	**L-asparagine**<br>**L-glutamine**<br>L-arginine	2
Adenosine	3	0.061	0.65	**5-deoxy-5-methylthioadenosine**<br>**S-5-adenosyl-L-homocysteine**<br>2-deoxyadenosine	2

Significant metabolites identified from the FC analysis (FC ≥ 1.2 or FC ≤ 0.83, and p-value ≤ 0.05) are presented in bold font; FDR: False Discovery Rate

Supplementary Table S4. Enrichment pathway analysis in the plasma from IP subjects using the central carbon metabolism intermediates.

Enriched pathways	Compounds	Hits	Enrichment ratio	p-value
Ammonia Recycling	32	14	2.1134	0.0071
Aspartate Metabolism	35	17	1.9054	0.0104
Pentose Phosphate Pathway	29	12	2.0232	0.0176
Pyrimidine Metabolism	59	25	1.5719	0.0276
Phenylacetate Metabolism	9	3	2.9638	0.0286
Fructose and Mannose Degradation	32	10	1.9365	0.0296
Warburg Effect	58	23	1.6003	0.0297
Spermidine and Spermine Biosynthesis	18	3	2.8165	0.0326
Histidine Metabolism	43	9	1.7994	0.0526
Estrone Metabolism	24	3	2.604	0.0539
Nicotinate and Nicotinamide Metabolism	37	12	1.7179	0.0542
Cysteine Metabolism	26	9	1.8057	0.0563
Alanine Metabolism	17	8	1.7691	0.0698
Glutathione Metabolism	21	6	1.8744	0.0749
Carnitine Synthesis	22	6	1.885	0.0754
Trehalose Degradation	11	3	2.2454	0.0769
Glycine and Serine Metabolism	59	19	1.4776	0.0777
Taurine and Hypotaurine Metabolism	12	2	2.5147	0.0787
Urea Cycle	29	13	1.5535	0.0817
Tryptophan Metabolism	60	12	1.5852	0.0873
Amino Sugar Metabolism	33	14	1.56	0.0878
Phosphatidylcholine Biosynthesis	14	6	1.7947	0.0886
Methionine Metabolism	43	12	1.5843	0.0889
Propanoate Metabolism	42	9	1.6073	0.0963
Glutamate Metabolism	49	19	1.4079	0.1009
Methylhistidine Metabolism	4	2	2.265	0.1025
Beta Oxidation of Very Long Chain Fatty Acids	17	3	2.04	0.1036
Phenylalanine and Tyrosine Metabolism	28	10	1.5677	0.1058
Arginine and Proline Metabolism	53	17	1.4085	0.1095
Lysine Degradation	30	7	1.6489	0.1101
Glycerol Phosphate Shuttle	11	4	1.8225	0.1201
Sphingolipid Metabolism	40	8	1.5483	0.1286
Mitochondrial Electron Transport Chain	19	7	1.5849	0.1293
Arachidonic Acid Metabolism	69	2	2.0275	0.1314
Oxidation of Branched Chain Fatty Acids	26	5	1.6774	0.1343
Androgen and Estrogen Metabolism	33	2	1.9931	0.1362
Androstenedione Metabolism	24	2	1.9931	0.1362
Ubiquinone Biosynthesis	20	2	1.982	0.1378
Porphyrin Metabolism	40	4	1.6991	0.1441
Glycolysis	25	11	1.4003	0.1583
Glucose-Alanine Cycle	13	5	1.5495	0.1688
Tyrosine Metabolism	72	9	1.41	0.1712
Vitamin B6 Metabolism	20	6	1.5001	0.1733
Catecholamine Biosynthesis	20	4	1.5818	0.1744
Glycerolipid Metabolism	25	6	1.4773	0.1774
Valine, Leucine and Isoleucine Degradation	60	14	1.3459	0.1785
Malate-Aspartate Shuttle	10	6	1.4702	0.1825
Biotin Metabolism	8	1	1.7951	0.1831
Phosphatidylinositol Phosphate Metabolism	17	4	1.5472	0.1852
Folate Metabolism	29	7	1.4106	0.1911
Pantothenate and CoA Biosynthesis	21	6	1.4354	0.1935
Purine Metabolism	74	34	1.2076	0.1936
Sulfate/Sulfite Metabolism	22	2	1.6522	0.1936

Enriched pathways	Compounds	Hits	Enrichment ratio	p-value
Lactose Degradation	9	2	1.6522	0.1936
Fatty acid Metabolism	43	5	1.4297	0.2087
Mitochondrial Beta-Oxidation of Short-Chain Saturated Fatty Acids	27	5	1.4297	0.2087
Mitochondrial Beta-Oxidation of Long-Chain Saturated Fatty Acids	28	5	1.4297	0.2087
Selenoamino Acid Metabolism	28	8	1.3448	0.2137
Thiamine Metabolism	9	4	1.4481	0.2155
Phosphatidylethanolamine Biosynthesis	12	6	1.3782	0.2183
Gluconeogenesis	35	15	1.2102	0.2532
Ethanol Degradation	19	3	1.2627	0.2897
Betaine Metabolism	21	6	1.2191	0.2935
Lactose Synthesis	20	8	1.1963	0.2973
Homocysteine Degradation	9	4	1.2013	0.3065
Threonine and 2-Oxobutanoate Degradation	20	7	1.1638	0.3225
Riboflavin Metabolism	20	5	1.1607	0.3304
Phytanic Acid Peroxisomal Oxidation	26	5	1.1415	0.3418
Bile Acid Biosynthesis	65	6	1.1251	0.3477
Mitochondrial Beta-Oxidation of Medium-Chain Saturated Fatty Acids	27	4	1.0831	0.3701
Beta-Alanine Metabolism	34	9	1.0665	0.3878
Steroid Biosynthesis	48	6	1.0225	0.4179
Butyrate Metabolism	19	5	0.9665	0.448
Starch and Sucrose Metabolism	31	10	0.9916	0.4545
D-Arginine and D-Ornithine Metabolism	11	1	0.5447	0.4664
Vitamin K Metabolism	14	1	0.5447	0.4664
Galactose Metabolism	38	12	0.947	0.4993
Cardiolipin Biosynthesis	11	4	0.8536	0.5022
Inositol Metabolism	33	5	0.8733	0.5063
Inositol Phosphate Metabolism	26	5	0.8733	0.5063
Citric Acid Cycle	32	13	0.9246	0.5302
Transfer of Acetyl Groups into Mitochondria	22	5	0.8146	0.549
Plasmalogen Synthesis	26	4	0.6662	0.6271
Pyruvate Metabolism	48	10	0.7918	0.6505
Nucleotide Sugars Metabolism	20	8	0.7339	0.6718
Fatty Acid Elongation In Mitochondria	35	1	0.1359	0.7167
Retinol Metabolism	37	1	0.1359	0.7167
Steroidogenesis	43	1	0.1359	0.7167
Caffeine Metabolism	24	2	0.3403	0.7183
Ketone Body Metabolism	13	2	0.3179	0.7359
Thyroid hormone synthesis	13	2	0.3008	0.7476
Pyruvaldehyde Degradation	10	2	0.2832	0.7581
Pterine Biosynthesis	29	2	0.1454	0.8697
Phospholipid Biosynthesis	29	3	0.2275	0.8833
De Novo Triacylglycerol Biosynthesis	9	2	0.0689	0.936

Significantly enriched pathways are in bold and illustrated in **Figure 4.**

Supplementary Table S5. Metabolites with good diagnostic value in IP and healthy subjects identified via biomarker analysis of plasma samples.

	AUC	p-value	log_2(FC)	Average Importance
trans-aconitic acid/2-deoxyuridine 5-triphosphate	0.8679	0.0002	-0.0251	2.0008
trans-aconitic acid/cytidine 5-triphosphate	0.8378	0.0001	-0.0217	2.2523
L-carnitine/trans-aconitic acid	0.8312	0.0001	-0.0304	2.1876
trans-aconitic acid/adenylosuccinic acid	0.8261	0.0001	-0.0216	2.1261
L-cystathionine/trans-aconitic acid	0.8245	0.0001	-0.0237	2.078
2-deoxycytidine 5-monophosphate/trans-aconitic acid	0.8211	0.0001	-0.023	2.2099
beta-nicotinamide adenine dinucleotide/trans-aconitic acid	0.8194	0.0001	-0.021	2.1791
4-hydroxyphenyl-pyruvic acid/trans-aconitic acid	0.8161	0.0001	-0.0206	2.1426
trans-aconitic acid/indoline-2-carboxylate	0.8161	0.0002	-0.0216	2.0629
5-hydroxy-3-indoleacetic acid/trans-aconitic acid	0.8061	0.0002	-0.0345	1.9135
trans-aconitic acid/deoxythymidine 5-triphosphate	0.8061	0.0001	-0.0206	2.1107
Argininosuccinic acid/trans-aconitic acid	0.8027	0.0001	-0.019	2.1177
Isopentenyl pyrophosphate/trans-aconitic acid	0.8027	0.0002	-0.0203	1.9894
L-homocysteine/trans-aconitic acid	0.8011	0.0001	-0.0191	2.1386
trans-aconitic acid	0.7977	0.0001	0.4768	2.0994
L-Canavanine/trans-aconitic acid	0.7927	0.0002	-0.0209	2.03
Folinic acid/trans-aconitic acid	0.7927	0.0001	-0.0197	2.146
Guanosine 3,5-cyclic monophosphate/trans-aconitic acid	0.791	0.0002	-0.02	2.0043
trans-aconitic acid/Flavin adenine dinucleotide	0.791	0.0002	-0.0193	2.0655
trans-aconitic acid/Inosine 5-triphosphate	0.786	0.0002	-0.0188	2.0242
2-Phosphoglyceric acid	0.7392	0.0145	0.1277	1.2953
L-asparagine	0.7342	0.0025	0.0898	1.6018
Malonic acid	0.7291	0.0074	-0.319	1.5203
DL-glyceraldehyde 3-phosphate	0.7024	0.0087	0.134	1.4071

AUC: Area under the ROC curve; FC: Fold change

Supplementary Table S6. LC-QToF MS acquired metabolites with good diagnostic value in IP and healthy subjects identified via biomarker analysis of plasma samples.

	AUC	p-value	log_2(FC)	Average Importance	IP
Unknown_102/2-Methoxy-3-(1-methylpropyl)pyrazine	0.8471	0.0004	-0.4093	1.9283	↑
Unknown_107/2-Methoxy-3-(1-methylpropyl)pyrazine	0.8326	0.0002	-1.3265	1.9889	↓
Unknown_13/Benzylazanium	0.8294	0.0004	0.2322	1.8076	↑
Unknown_107/Pirimicarb	0.8213	0.0001	1.4711	2.1637	↑
2,4-Diaminotoluene/Vanillylamine	0.8197	0.0001	0.5654	2.0941	↓
Unknown_40/Vanillylamine	0.8084	0.0001	1.1964	2.1058	↓
Unknown_35/Unknown_107	0.8036	0.0003	-1.0187	1.9948	↓
Unknown_59/Unknown_107	0.8036	0.0003	-0.4558	1.9327	↓
Unknown_107/Cinnamyl cinnamate	0.8020	0.0002	-0.5846	2.0009	↑
11-amino-undecanoic acid/Vanillylamine	0.8020	0.0001	0.3974	2.2121	↑
Unknown_107/Dimethylenetriurea	0.7988	0.0002	-99	2.0268	↑
Benzylazanium/Butyric acid	0.7972	0.0001	0.1325	2.0529	↑
Unknown_28/Benzylazanium	0.7827	0.0002	0.2676	1.9679	↓
Unknown_107/11-amino-undecanoic acid	0.7810	0.0003	-1.7056	2.0028	↑
Unknown_107/Hippuric acid	0.7794	0.0003	-1.1011	1.9523	↓
Isoamyl nitrite/Vanillylamine	0.7746	0.0002	0.3076	1.9452	↓
Unknown_107/Methylenediurea	0.7730	0.0004	-99	1.9197	↓
2-Methoxy-3-(1-methylpropyl)pyrazine	0.7698	0.0412	0.0272	1.1161	↑
Unknown_40/Unknown_107	0.7682	0.0001	-0.5697	2.0508	↑
Unknown_22/Benzylazanium	0.7649	0.0002	0.2593	1.9321	↓
Unknown_13	0.7601	0.0017	-0.021	1.6119	↑
Unknown_107	0.7472	0.0011	-0.0703	1.7873	↑
(S)-3-Octanol glucoside	0.7166	0.0204	-0.0293	1.1805	↓
Unknown_8	0.7070	0.0168	-0.0591	1.4671	↑

AUC: Area under the ROC curve; FC: Fold change; The arrows in the pre-LG column indicate the levels (↑ - high and ↓ - low) in the IP subjects.

Supplementary Table S7. LC-QToF MS acquired lipids with good diagnostic value in IP and healthy subjects identified via biomarker analysis of plasma samples.

	AUC	p-value	log₂(FC)	Average Importance	IP
Cer_NS d18:2_24:0/TG 15:0_16:0_18:2	0.8452	0.0001	1.0859	2.0695	↓
Cer_NS d18:2_24:0/TG 15:0_16:1_18:2	0.8452	0.0001	-99	2.117	↓
Cer_NS d18:1_24:1/TG 15:0_16:0_18:2	0.8435	0.0001	1.106	1.9621	↓
ACar 12:1/SM d36:2	0.8384	0.0001	0.2137	1.9458	↑
Cer_NS d18:1_24:1/TG 15:0_16:1_18:2	0.8384	0.0001	-99	1.9824	↑
ACar 12:1/TG 15:0_16:1_18:2	0.8351	0.0001	0.8537	1.8546	↑
TG 15:0_16:0_18:2/TG 16:0_18:1_18:2	0.83	0.0001	-0.2904	1.964	↑
TG 15:0_16:0_18:2/TG 52:3	0.83	0.0001	-0.2907	1.9663	↓
ACar 12:1/TG 15:0_16:0_18:2	0.8283	0.0001	0.5204	1.8982	↓
ACar 12:1/LPC 15:0/0:0	0.8199	0.0001	-99	1.8394	↑
ACar 10:0/TG 15:0_16:0_18:2	0.8182	0.0001	0.8285	1.7832	↑
ACar 12:1/PC 14:0_16:0	0.8149	0.0001	0.4366	1.7754	↑
TG 15:0_16:0_18:1/TG 16:0_18:1_18:2	0.8081	0.0001	-0.3049	1.8915	↓
TG 15:0_16:0_18:1/TG 52:3	0.8081	0.0001	-0.3051	1.8933	↓
TG 16:0_17:0_18:1/TG 16:0_18:1_18:2	0.8064	0.0001	-0.3579	1.8673	↓
TG 16:0_17:0_18:1/TG 52:3	0.8064	0.0001	-0.3582	1.8691	↓
ACar 12:1/TG 15:0_16:0_18:1	0.8048	0.0001	0.5339	1.7776	↓
TG 51:3/TG 54:4	0.8014	0.0001	-0.3314	1.7629	↑
TG 15:0_18:1_18:2/TG 54:4	0.7997	0.0001	-0.3313	1.763	↓
PC 16:0_16:1	0.7846	0.0124	0.0639	1.3381	↑
PC 33:1	0.7812	0.0036	0.0472	1.394	↓
TG 15:0_16:0_18:2	0.7778	0.0006	0.068	1.6014	↓
TG 15:0_16:1_18:2	0.7694	0.0013	0.0695	1.5192	↓
TG 16:0_17:0_18:1	0.7576	0.0011	0.0779	1.5431	↓
TG 51:1	0.7559	0.0014	0.0744	1.515	↑
TG 15:0_16:0_18:1	0.7526	0.0011	0.0715	1.5322	↓
PC 31:1	0.7357	0.0363	0.1141	1.0549	↓
LPC 15:0/0:0	0.7341	0.0051	0.0386	1.2222	↓
TG 16:0_17:1_18:1	0.7341	0.0018	0.0535	1.4771	↓
TG 51:2	0.7341	0.0018	0.0534	1.4766	↓
PE 16:0_20:4	0.729	0.0158	0.0449	1.2705	↑
PE 16:0_18:2	0.7256	0.0173	0.0373	1.265	↑
TG 15:0_18:1_18:2	0.7223	0.0084	0.0365	1.2619	↑
TG 51:3	0.7223	0.0084	0.0365	1.2619	↓
ACar 10:0	0.7206	0.0126	-0.0452	1.306	↑
PC 17:0_18:1	0.7155	0.0302	0.0623	1.1142	↓
PE 18:0_20:4	0.7155	0.0108	-0.0523	1.3976	↓
TG 14:0_16:0_16:0	0.7155	0.0134	0.0428	1.2185	↓
TG 16:0_16:1_18:1	0.7155	0.0078	0.0267	1.2614	↓
TG 50:2	0.7155	0.0079	0.0267	1.2599	↓
PC 14:0_14:0	0.7122	0.0143	0.0665	1.125	↑
TG 48:2	0.7122	0.0048	0.056	1.4056	↓
PC 14:0_16:0	0.7105	0.0147	0.0383	1.1359	↑
LPC 0:0/14:0	0.7004	0.0155	0.0334	1.1004	↑

AUC: Area under the ROC curve; FC: Fold change; The arrows in the IP column indicate the levels (↑ - high and ↓ - low) in the IP subjects.
